# Predictors of social participation: evidence from repeated cross-sectional population surveys in England

**DOI:** 10.1093/pubmed/fdac029

**Published:** 2022-03-17

**Authors:** A Wilding, L A Munford, M Sutton

**Affiliations:** Health Organisation, Policy and Economics, School of Health Sciences, University of Manchester, Manchester M13 9PL, UK; Health Organisation, Policy and Economics, School of Health Sciences, University of Manchester, Manchester M13 9PL, UK; Health Organisation, Policy and Economics, School of Health Sciences, University of Manchester, Manchester M13 9PL, UK; Melbourne Institute: Applied Economic and Social Research, University of Melbourne, Melbourne, VIC 3010, Australia

**Keywords:** community assets, predictors, social participation

## Abstract

**Background:**

Social participation is linked to better health and well-being. However, there is limited research on the individual and area-level predictors of participation. This study aims to determine the characteristics associated with participation, particularly the impact of community asset availability.

**Methods:**

We used data from 34 582 adult respondents to the nationally representative Community Life Survey from 2013 to 2018. We measured social participation by reported participation in 15 types of groups. We used probit and negative binomial regression models and included a wide range of individual, household and area characteristics, and availability of 14 types of community assets.

**Results:**

The following characteristics were associated with higher levels of participation: being female (+3.0 percentage points (p.p.) (95% CI 1.8 to 4.1p.p.), Black, Asian or Minority Ethnicity (+3.7p.p. (1.9 to 5.5p.p.)), homeownership (+4.1 p.p. (2.7 to 5.6p.p.)) and living in a rural area (+2.1p.p. (0.5 to 3.6p.p)). Respondents from the most deprived areas were less likely to participate than those in average deprivation areas (−3.9p.p. (−5.9 to −1.99p.p.)). Higher availability of community assets was associated with increased participation in groups. The effect of availability on participation varied by type of asset.

**Conclusion:**

Improving community assets infrastructure in high deprivation and urban areas would encourage more social participation in these areas.

## Introduction

Social participation is defined as an individual’s involvement in activities that provide connections with others in the society or community,^1^ which can be through a specific activity or by volunteering.^2^ The key component is the interaction with others, with past research demonstrating links between social participation and reduced social isolation,^3^ and improved health and well-being.^4^

Current policies, such as ‘social prescribing’ in the UK, demonstrate how social participation acts as a complement or substitute to formal healthcare, as individuals are prescribed to attend social groups in addition to, or instead of, medical treatments.^5^ Individuals who engage with social prescribing have been shown to have improved health and well-being,^6^ with men having higher potential gains than women.^7^ For these policies to be effective and have longevity, they require, firstly high levels of participation from individuals across all population strata,^8^ and secondly good access to facilities for these groups.^9^ Such community assets include community centres, parks and leisure facilities.^10^ Past research has shown that participating in community assets improved quality of life, reduced health care costs^4^ and reduced mortality.^11^ In recent years, there has been additional funding of community assets in the UK,^12^^,^^13^ with the aim to protect local facilities used by the community to act as hubs for social participation.

Prior to the implementation of such policies, it is important to understand who currently participates in these facilities and services because those who choose to participate, in the absence of policy encouragement, may have different characteristics to those who do not. Determining those characteristics can inform policy around who to target. There are some known factors that enable, and others that act as barriers to, participation. For example, rurality acts as a barrier, and educational attainment, income, ethnicity and deprivation have been shown to be predictive in non-UK studies of older people.^14^ However, much less is known about the predictors of social participation in a general population sample of all ages. This is important because it is often assumed that continued participation across the life course will produce greater returns to health.^11^ Past research has also failed to assess the role of the local infrastructure of community assets on participation. For example, if assets are not available close to where an individual lives, this could act as a barrier. Further understanding of how community assets facilitate participation is required.

In this paper, we use data from a large sample of respondents to repeated cross-sectional national surveys to: (i) assess the predictors of social participation in England amongst individuals aged over 16; and (ii) estimate the extent to which the availability of community assets enables social participation.

## Methods

### Data

We obtained data from the 2013 to 2018 waves of the Community Life Survey. This is a random stratified cross-sectional sample of English individuals aged over 16 years.^15^ The data were collected by three modes in 2013 and 2015: online; postal; and face-to-face. Only the online and face-to-face modes were used in 2014 and only the online and postal modes were used in 2016, 2017 and 2018. We used only the online responses for three reasons. Firstly, these 36 768 observations account for the majority (64.5%) of responses over these years. Secondly, we excluded the postal questionnaires as they did not contain the key variables of community asset availability. Finally, the survey provides separate sample weights for: (a) online responses only; (b) online and postal responses combined and (c) face-to-face responses only. We were unable to combine the weights for the online and face-to-face responses due to the broad regional identifiers used for re-calculation. Therefore, to ensure the weighted estimation sample was representative of England’s population, we used online responses only.

The Community Life Survey contains questions on *participation* in groups and *availability* of community assets. Individuals are asked to report their participation in a pre-specified list of 15 types of groups. The complete list of groups is provided in the Supplementary Appendix. We created three binary variables based on responses to these questions: (1) participation in any group; (2) participation in a sports group, as this had the highest participation rate and (3) participation in any group except sports. We additionally created a variable equal to the count of the number of groups that an individual reported participating in.

Respondents were also asked which assets from a pre-specified list of 14 were available within a 15-to-20-min walk. We created two measures of availability: (1) a series of binary variables equal to one if an individual reported each asset type was available within a 15-to-20-min walk; and (2) the count of all community assets reported as being within a 15-to-20-min walk.

We included the following individual characteristics: age in 8-year bands, gender, ethnicity, educational attainment and marital status. We also included the following household-level characteristics: housing tenure, household composition, and log-transformed equivalized real household income (which we generated via an interval regression model from the reported categorical bands—see Supplementary Appendix). We included the following area-level characteristics: Government Office Region; quintiles of the Index of Multiple Deprivation^16^; and urban–rural classification. To account for time trends, we included binary variables for each survey year. We include in the Supplementary Appendix a detailed table of these variables.

### Statistical analysis

We carried out the analysis using STATA v16 for Mac.^17^ We used binary probability unit (probit) models to determine the individual, household and area-level predictors associated with the outcomes. For the count outcomes, we used a negative binomial regression. For both models, we used robust standard errors and adjusted to England’s population using probability weights. Post estimation, we calculated average marginal effects (AME) to summarize the effects of each variable on the outcome. For each region, we report the deviation contrast, representing differences from a grand mean.^18^

To assess whether asset availability predicted participation, we estimated the models with two measures of availability and the remaining characteristics acting as covariates. The two measures were estimated separately for each outcome. Sensitivity analysis was conducted to assess whether it was the count of the number of assets available or the separate availabilities of each type of asset that best predicted participation. We used the Bayesian Information Criterion (BIC)^19^ for this purpose, with the smallest value indicating the better fit for that particular outcome.

## Results

### Descriptive statistics

The full sample was 36 768. We excluded 1 286 individuals (3.5%) due to missing values in either the outcomes or the key independent variables. 35 482 individuals were included in the estimation sample.

Table 1 contains descriptive statistics for the participation outcomes. 25 937 individuals (73.1%) participated in at least one group. The average number of groups that an individual participated in was approximately two. Sport groups were the most commonly participated in (41.6%). When excluding these groups, 64.8% of the sample participated in at least one other group. Approximately a quarter of the sample participated in either hobbies & arts and/or religious groups.

**Table 1:** Descriptive statistics of participation in social groups

	Mean	*n*/(sd)
Participation in any group	0.731	25 951
Participation in any group except Sports	0.648	23 004
Number of groups participated in (out of 14)	1.998	(1.986)
**Which groups?**		
Sport/exercise	0.416	14 762
Local community	0.119	4221
Religion	0.247	8763
Hobbies & arts	0.271	9609
Children’s education	0.156	5531
Citizens	0.045	1579
Environment	0.135	4805
Safety & first aid	0.058	2044
Education for adults	0.104	3688
Children’s activities	0.126	4483
The elderly	0.082	2917
Justice & human rights	0.037	1321
Politics	0.040	1420
Health & social welfare	0.108	3839
Trade union	0.077	1901
Observations	35 482	

Descriptive statistics of the predictors and asset availability are reported in Table 2, stratified by participation status. There are significant differences in predictors by participation status, particularly for educational attainment. The differences are also significant by gender, ethnicity, household income, household composition, housing tenure, deprivation and rurality. The differences were mixed for age, marital status and region. Across the indicators of asset availability, there were significant differences across all types of asset except general shops, indicating that participants had improved availability.

**Table 2:** Descriptive statistics of predictors of participation, stratified by participation status

	Participant		Non-participant		Difference (Participant–non-participant)		
	Mean	*n*/(sd)	Mean	*n*/(sd)	Diff	*t*-stat	*P*-value
**Individual**							
Female	0.532	13 813	0.513	4891	0.019	3.195	0.001
**Age (in years)**							
16–19	0.039	1000	0.037	355	0.001	0.561	0.575
20–24	0.048	1258	0.069	655	−0.020	−7.491	<0.001
25–34	0.149	3862	0.190	1815	−0.042	−9.489	<0.001
35–49	0.275	7131	0.243	2320	0.031	5.928	<0.001
50–64	0.263	6837	0.263	2506	0.001	0.100	0.920
65–69	0.092	2378	0.084	797	0.008	2.344	0.019
70–74	0.069	1778	0.054	512	0.015	5.029	<0.001
75+	0.066	1707	0.060	571	0.006	1.999	0.046
Black, Asian or Minority Ethnicity	0.150	3883	0.157	1496	−0.007	−1.707	0.088
**Highest qualification obtained**							
No qualifications	0.066	1473	0.169	1425	−0.103	−28.069	<0.001
GCSEs	0.248	5565	0.330	2784	−0.082	−14.491	<0.001
A Levels	0.189	4248	0.186	1569	0.003	0.674	<0.001
Degree	0.498	11 180	0.316	2670	0.182	28.997	<0.001
**Marital status**							
Single	0.266	6909	0.323	3076	−0.057	−10.507	<0.001
Married	0.563	14 602	0.488	4647	0.075	12.615	<0.001
Divorced/Separated	0.107	2784	0.112	1067	−0.005	−1.254	0.210
Widowed	0.041	1066	0.042	400	−0.001	−0.374	0.709
Missing	0.023	590	0.036	341	−0.013	−6.817	<0.001
**Household**							
Logged income	9.485	(0.925)	9.274	(0.915)	0.211	19.129	<0.001
Number of adults	2.216	(0.940)	2.271	(0.983)	−0.056	−4.880	<0.001
Number of children	0.487	(0.852)	0.396	(0.784)	0.090	9.020	<0.001
Homeowner	0.710	(0.454)	0.584	18429	0.126	22.692	<0.001
**Area deprivation quintile**							
1—Most deprived	0.152	3957	0.224	2139	−0.072	−15.981	<0.001
2	0.181	4709	0.220	2093	−0.038	−8.097	<0.001
3	0.201	5223	0.197	1878	0.004	0.881	0.378
4	0.218	5663	0.183	1741	0.036	7.310	<0.001
5—Least deprived	0.247	6399	0.176	1680	0.070	14.038	<0.001
**Region**							
North East	0.035	919	0.042	403	−0.007	−3.029	0.002
North West	0.114	2956	0.122	1166	−0.008	−2.197	0.028
Yorkshire and Humberside	0.087	2268	0.095	910	−0.008	−2.363	0.018
East Midlands	0.076	1976	0.086	815	−0.009	−2.905	0.004
West Midlands	0.096	2487	0.099	945	−0.003	−0.937	0.349
East of England	0.115	2981	0.112	1067	0.003	0.767	0.443
London	0.190	4935	0.191	1825	−0.001	−0.279	0.780
South East	0.185	4795	0.164	1559	0.021	4.618	<0.001
South West	0.101	2634	0.088	841	0.013	3.726	<0.001
**Rural**	0.185	4803	0.144	1370	0.041	9.115	<0.001
**Availability**							
Number of assets in 15–20-min walk (out of 14)	10.526	(3.123)	10.057	(3.280)	0.468	12.350	<0.001
**Asset within 15–20 min:**							
Pub	0.892	23 158	0.862	8218	0.030	7.872	<0.001
Sports centre	0.547	14 208	0.484	4612	0.064	10.656	<0.001
Place of worship	0.869	22 549	0.803	7655	0.066	15.476	<0.001
Park	0.879	22 809	0.855	8149	0.024	5.992	<0.001
Community centre	0.678	17 588	0.594	5664	0.083	14.707	<0.001
Public transport	0.933	24 225	0.910	8677	0.023	7.431	<0.001
Post office	0.821	21 305	0.803	7657	0.018	3.793	<0.001
Secondary school	0.574	14 889	0.556	5302	0.017	2.941	0.003
Youth club	0.314	8137	0.263	2510	0.050	9.156	<0.001
Library	0.633	16 428	0.606	5777	0.027	4.644	<0.001
General shop	0.921	23 898	0.917	8739	0.004	1.226	0.220
Primary school	0.878	22 779	0.841	8019	0.036	8.990	<0.001
Health centre	0.778	20 184	0.757	7213	0.021	4.177	<0.001
Chemist	0.809	20 993	0.804	7664	0.005	1.024	0.306
Observations	25 951		9531		35 482		

### Determinants of participation

Table 3 reports the results for predictors of participation. Individuals aged over 50 have increased rates of participation in any group, particularly those aged 70–74 years with a 19.4 percentage point (CI = 0.172–0.198) increase compared to those age 35–49 years.

**Table 3:** Predictors of group participation

	Participation in any group	Participation in any group except Sports groups	Participation in sports group	Number of groups participated in
	AME (95% CI)	AME (95% CI)	AME (95% CI)	AME (95% CI)
**Age (Refs:** 35–49**)**				
16–19	0.142^***^	0.129^***^	0.177^***^	0.814^***^
	(0.113 to 0.172)	(0.093 to 0.164)	(0.138 to 0.215)	(0.595 to 1.032)
20–24	−0.007	−0.030	0.044^**^	0.098
	(−0.038 to 0.024)	(−0.063 to 0.002)	(0.012–0.077)	(−0.058 to 0.254)
25–34	−0.040^***^	−0.064^***^	−0.024^*^	−0.205^***^
	(−0.059 to −0.020)	(−0.084 to −0.045)	(−0.043 to −0.005)	(−0.274 to −0.137)
50–64	0.019^*^	0.029^**^	−0.028^**^	0.112^**^
	(0.002 to 0.036)	(0.011 to 0.046)	(−0.045 to −0.010)	(0.044 to 0.179)
65–69	0.060^***^	0.073^***^	−0.011	0.422^***^
	(0.038 to 0.083)	(0.049 to 0.097)	(−0.036 to 0.013)	(0.318 to 0.527)
70–74	0.194^***^	0.242^***^	0.111^***^	1.871^***^
	(0.172 to 0.216)	(0.217 to 0.266)	(0.075 to 0.146)	(1.549 to 2.193)
75+	0.174^***^	0.235^***^	0.026	1.786^***^
	(0.149 to 0.198)	(0.209 to 0.262)	(−0.011 to 0.063)	(1.464 to 2.108)
Female	0.030^***^	0.062^***^	−0.035^***^	0.223^***^
	(0.018 to 0.041)	(0.050 to 0.074)	(−0.047 to −0.023)	(0.174 to 0.272)
BAME	0.037^***^	0.070^***^	−0.047^***^	0.214^***^
	(0.019 to 0.055)	(0.051 to 0.089)	(−0.067 to −0.028)	(0.126 to 0.302)
**Marital status (Ref: Married)**				
Single	−0.018^*^	−0.042^***^	0.015	−0.079^*^
	(−0.035 to −0.002)	(−0.060 to −0.024)	(−0.003 to 0.034)	(−0.158 to −0.001)
Divorced/separated	−0.013	−0.026^*^	0.021	0.022
	(−0.033 to 0.007)	(−0.047 to −0.005)	(−0.000 to 0.042)	(−0.072 to 0.115)
Widowed	−0.021	−0.015	−0.040^*^	−0.090
	(−0.054 to 0.011)	(−0.049 to 0.020)	(−0.075 to −0.005)	(−0.224 to 0.044)
Not reported	−0.049^**^	−0.047^*^	−0.048^*^	−0.105
	(−0.084 to −0.015)	(−0.084 to −0.010)	(−0.086 to −0.011)	(−0.272 to 0.063)
**Highest qual (No education)**				
GCSEs	0.120^***^	0.115^***^	0.102^***^	0.443^***^
	(0.097 to 0.144)	(0.091 to 0.138)	(0.078 to 0.126)	(0.321 to 0.566)
A Levels	0.202^***^	0.200^***^	0.168^***^	0.850^***^
	(0.178 to 0.226)	(0.175 to 0.225)	(0.142 to 0.194)	(0.707 to 0.993)
Degree	0.250^***^	0.273^***^	0.195^***^	1.333^***^
	(0.228 to 0.273)	(0.250 to 0.296)	(0.170 to 0.220)	(1.182 to 1.484)
Logarithmic income	0.028^***^	0.003	0.057^***^	0.051^*^
	(0.019 to 0.037)	(−0.007 to 0.013)	(0.048 to 0.067)	(0.006 to 0.095)
# of Adults	0.004	−0.004	0.018^***^	0.027
	(−0.004 to 0.011)	(−0.012 to 0.004)	(0.009 to 0.026)	(−0.016 to 0.070)
# of Children	0.038^***^	0.052^***^	0.033^***^	0.259^***^
	(0.030 to 0.047)	(0.043 to 0.061)	(0.024 to 0.042)	(0.220 to 0.298)
Homeowner	0.041^***^	0.031^***^	0.053^***^	0.232^***^
	(0.027 to 0.056)	(0.016 to 0.047)	(0.037 to 0.069)	(0.157 to 0.307)
**Area deprivation quintile**				
1—Most	−0.039^***^	−0.028^**^	−0.048^***^	−0.102^*^
	(−0.059 to −0.019)	(−0.050 to −0.007)	(−0.069 to −0.027)	(−0.191 to −0.013)
2	−0.022^*^	−0.014	−0.013	−0.050
	(−0.040 to −0.004)	(−0.033 to 0.006)	(−0.032 to 0.006)	(−0.127 to 0.027)
4	0.023^**^	0.017	0.045^***^	0.117^**^
	(0.006 to 0.040)	(−0.001 to 0.035)	(0.027 to 0.063)	(0.043 to 0.191)
5—Least	0.036^***^	0.037^***^	0.059^***^	0.164^***^
	(0.019 to 0.052)	(0.019 to 0.055)	(0.040 to 0.077)	(0.093 to 0.235)
**Region**				
North East	−0.028	−0.031^*^	−0.019	−0.148^*^
	(−0.056 to 0.000)	(−0.061 to −0.002)	(−0.048 to 0.011)	(−0.270 to −0.026)
North West	0.003	0.002	0.032^***^	0.047
	(−0.012 to 0.018)	(−0.014 to 0.018)	(0.016 to 0.049)	(−0.023 to 0.116)
Yorkshire and Humberside	0.000	−0.014	0.016	−0.093^**^
	(−0.017 to 0.017)	(−0.032 to 0.005)	(−0.003 to 0.034)	(−0.163 to −0.023)
East Midlands	−0.012	−0.015	−0.017	−0.053
	(−0.030 to 0.007)	(−0.034 to 0.005)	(−0.038 to 0.003)	(−0.138 to 0.032)
West Midlands	−0.002	0.001	−0.014	−0.037
	(−0.018 to 0.015)	(−0.016 to 0.019)	(−0.032 to 0.004)	(−0.111 to 0.036)
East of England	−0.018^*^	−0.020^*^	−0.012	−0.076^*^
	(−0.033 to −0.002)	(−0.036 to −0.004)	(−0.028 to 0.004)	(−0.140 to −0.013)
London	0.015^*^	0.029^***^	−0.015^*^	0.093^**^
	(0.001 to 0.028)	(0.015 to 0.044)	(−0.030 to −0.001)	(0.030 to 0.155)
South East	−0.000	−0.008	0.003	0.007
	(−0.012 to 0.012)	(−0.021 to 0.005)	(−0.010 to 0.016)	(−0.044 to 0.058)
South West	0.018^*^	0.026^**^	0.013	0.115^**^
	(0.002 to 0.034)	(0.009 to 0.043)	(−0.005 to 0.030)	(0.040 to 0.191)
Rural LSOA	0.021^**^	0.034^***^	0.006	0.210^***^
	(0.005 to 0.036)	(0.018 to 0.050)	(−0.011 to 0.022)	(0.147 to 0.273)
**Survey year (Ref: 2013)**				
2014	−0.037^**^	−0.037^**^	−0.050^***^	−0.177^***^
	(−0.061 to −0.013)	(−0.062 to −0.011)	(−0.076 to −0.024)	(−0.282 to −0.072)
2015	−0.046^***^	−0.043^**^	−0.047^***^	−0.115
	(−0.071 to −0.022)	(−0.069 to −0.017)	(−0.074 to −0.020)	(−0.245 to 0.016)
2016	−0.055^***^	−0.058^***^	−0.040^***^	−0.216^***^
	(−0.072 to −0.038)	(−0.077 to −0.040)	(−0.058 to −0.021)	(−0.294 to −0.137)
2017	−0.065^***^	−0.075^***^	−0.038^***^	−0.271^***^
	(−0.082 to −0.049)	(−0.093 to −0.058)	(−0.056 to −0.020)	(−0.346 to −0.195)
2018	−0.077^***^	−0.084^***^	−0.053^***^	−0.354^***^
	(−0.093 to −0.061)	(−0.101 to −0.067)	(−0.071 to −0.036)	(−0.426 to −0.283)
Observations	35 482	35 482	35 482	35 482

Note: 95% CI based on robust standard errors in parentheses, ^*^P < 0.05, ^**^P < 0.01, ^***^P < 0.001, probability weights are used to adjust to population of England. Average marginal effect (AME), confidence interval (CI). Base categories in brackets.

Women have higher rates of participation in any group by 3.0 percentage points (CI = 0.018–0.041). Individuals who are Black, Asian or Minority Ethnicity have a 3.7 percentage point higher probability of participating in any group (CI = 0.019–0.055) than White respondents.

Educational attainment is a predictor of participation across all outcomes, with incremental increases across each level. Those with a degree have increased probability of participation by 25.0 percentage points (CI = 0.228–0.273) compared to those who have no qualification.

Household income is a predictor of participation in any group, with a one percent increase in income increasing participation in any group by 2.8 percentage points (CI = 0.019–0.037).

Individuals living in households with dependents have increased participation across all outcomes. Housing tenure predicts participation, with those owning their own home having increased participation in any group by 4.1 percentage points (CI = 0.027–0.056).

Conditional on these individual- and household-level socioeconomic characteristics, the deprivation level of the area in which an individual lives also predicts participation. Those living in the most deprived areas have a reduced probability of participation in all the binary outcomes compared to the middle deprivation level. Those who live in the least deprived areas are significantly more likely to participate and attended more groups, on average.

Compared to the national average, those who live in London have increased probability of participating in all groups, except sports groups where this effect is negative and significant. Individuals who live in the North West have increased rates of participating in sports groups, with areas including East of England and the North East having reduced rates of participating in groups and the South West having increased rates of participation.

Those who reside in rural areas have increased participation in any groups by 2.1 percentage points (CI = 0.005–0.036), particularly non-sport groups by 3.4 percentage points (CI = 0.018–0.050). There are no significant differences in participation in sports groups between urban and rural areas.

### The effect of availability of community assets on participation

The conditional associations between availability of community assets and participation are reported in Table 4. When participation in any group is the outcome, the BIC value is smaller for the model where the count of available assets is included compared to the series of binary indicators of whether each type of asset is available. This model shows that participation increases by 0.7 (CI = 0.5–0.8) percentage points for each additional asset available. The same is true for participation in any group except sports groups, where participation increases by 0.6 (CI = 0.4–0.8) percentage points per asset available.

**Table 4:** Effects of asset availability on social participation

	Participation in any group	Participation in any group except Sports groups	Participation in sports group	Number of groups participated in
	AME	AME	AME	AME
	(95% CI)	(95% CI)	(95% CI)	(95% CI)
**Count**				
Assets	0.007^***^	0.006^***^	0.009^***^	0.028^***^
	(0.005 to 0.008)	(0.004 to 0.008)	(0.007 to 0.011)	(0.018 to 0.037)
BIC	41 261.2	45 530.5	47 150.2	132 589.7
**Individual**				
Pub	−0.010	−0.028**	0.018	−0.153**
	(−0.029 to 0.010)	(−0.049 to −0.007)	(−0.004 to 0.039)	(−0.254 to −0.053)
Sports centre	0.029^***^	0.014^*^	0.073^***^	0.148^***^
	(0.017 to 0.042)	(0.000 to 0.028)	(0.059 to 0.087)	(0.090 to 0.205)
Place of worship	0.046^***^	0.052^***^	0.027^**^	0.272^***^
	(0.028 to 0.063)	(0.033 to 0.072)	(0.007 to 0.048)	(0.188 to 0.357)
Park	0.013	0.009	0.015	0.038
	(−0.006 to 0.032)	(−0.011 to 0.029)	(−0.006 to 0.036)	(−0.051 to 0.127)
Community centre	0.021^**^	0.018^*^	0.011	0.149^***^
	(0.008 to 0.034)	(0.004 to 0.033)	(−0.003 to 0.026)	(0.088 to 0.211)
Public transport	0.013	−0.003	0.012	−0.189^**^
	(−0.011 to 0.036)	(−0.029 to 0.022)	(−0.014 to 0.039)	(−0.309 to −0.069)
Post office	−0.002	0.003	−0.006	−0.020
	(−0.019 to 0.015)	(−0.015 to 0.021)	(−0.025 to 0.013)	(−0.101 to 0.062)
Secondary school	−0.011	−0.016^*^	0.003	−0.050
	(−0.024 to 0.002)	(−0.030 to −0.002)	(−0.011 to 0.017)	(−0.107 to 0.008)
Youth club	0.021^**^	0.032^***^	0.014	0.202^***^
	(0.007 to 0.035)	(0.017 to 0.047)	(−0.001 to 0.029)	(0.143 to 0.262)
Library	−0.004	0.011	−0.019^*^	0.072^*^
	(−0.018 to 0.010)	(−0.004 to 0.026)	(−0.035 to −0.004)	(0.007 to 0.136)
General shop	−0.022	−0.018	−0.020	−0.141^*^
	(−0.046 to 0.002)	(−0.044 to 0.007)	(−0.046 to 0.006)	(−0.259 to −0.024)
Primary school	0.008	0.014	−0.019	−0.031
	(−0.012 to 0.027)	(−0.007 to 0.035)	(−0.041 to 0.002)	(−0.126 to 0.064)
Health centre	0.006	−0.000	0.008	−0.007
	(−0.012 to 0.023)	(−0.019 to 0.019)	(−0.012 to 0.027)	(−0.088 to 0.073)
Chemist	−0.023^*^	−0.024^*^	−0.003	−0.140^**^
	(−0.043 to −0.003)	(−0.045 to −0.003)	(−0.025 to 0.018)	(−0.231 to −0.048)
BIC	41 277.6	45 544.2	47 110.0	132 395.9
Observations	35 482	35 482	35 482	35 482

Note: 95% CI based on robust standard errors in parentheses, ^*^P < 0.05, ^**^P < 0.01, ^***^P < 0.001, Models also contain the following covariates: gender, age, ethnicity, marital status, education attainment, household income, household composition, housing tenure, index of multiple deprivation quintile, government office region, rural–urban classification and sample year. Probability weights are used to adjust to population of England. Average marginal effect (AME), beta (B), confidence interval (CI), Bayesian Information Criterion (BIC).

For participation in sport groups, the BIC estimator is smallest in the model where each individual asset is included. Having a sports centre available increases the probability of participation by 7.3 (CI = 5.9–8.7) percentage points and participation is 2.7 (CI = 0.7–4.8) percentage points higher if a place of worship is available. For the number of groups attended, the BIC value indicated the inclusion of individual assets is preferred. Having places of worships available has the largest effect on number of groups participated in (AME = 0.272 CI = 0.188–0.357), along with sports centres, community centres, youth clubs and libraries. The availability of a pubs, general shops, public transport and chemists decreased the number of groups participated in.

## Discussion

### Main findings of this study

We investigated the predictors of social participation for adults in England using a nationally representative sample of the general population. We found that the key predictors of participation were educational attainment—particularly having a degree—gender, and ethnicity. Household characteristics also predicted participation, with being a homeowner being the most important factor. Area-level characteristics also predicted participation, with lower rates in deprived areas and higher rates in the least deprived areas. There was more participation in the South compared to the North, particularly in London. Those who lived in rural areas had higher participation compared to urban dwellers.

The availability of community assets was associated with increased participation in groups. A high concentration of different assets was key to predicting participation in any group. The availabilities of different types of assets predicted participation better for sports groups and for the total number of groups that an individual participated in.

### What is already known

This study supports the findings of a past systematic review on predictors of social participation,^14^ with educational attainment, income and area deprivation predicting participation. However, this study finds different results in terms of minority ethnicity and rurality. We find those characteristics were associated with higher participation, whereas past research found the opposite. These results could highlight difference across countries or in the behaviours of younger adults. With past research focusing predominately on older populations, this study provides insight into those who currently participate across age strata, which is important because continued participation across life stages generates greater returns to health.^11^ The results found for deprivation level supports the need to encourage participation across the population strata,^8^ as currently those who participate are more likely to be living in the least deprived areas which could contribute to existing health inequalities.^20^^,^^21^

The predictors of participation are characteristics that are difficult for individuals to change to aid participation; however, the availability of community assets is easier to alter via building infrastructure through existing policies like the community renewal fund.^13^ The results of this study demonstrates that availability enables participation, be that a concentration of assets to stimulate some participation or availability of specific types of assets to encourage participation in sports and a larger number of groups. These findings are supported by past research showing that individuals need access to participate.^9^ We find that availability of pubs, general shops, public transport and chemists reduces the number of groups participated in by individuals. The negative coefficient on pubs can be explained from past findings that concluded that pubs were alternative ways to create community cohesion and social interactions.^22^ Past research in the UK found that those who live in the most deprived areas have improved access to chemists.^23^ Our finding that better access to these facilities is associated with reduced rates of social participation may reflect that participation in formal social groups is lower in areas with better transport and retail infrastructure.

### What this study adds

The use of data from large, nationally representative surveys provides insight into who currently engages in social participation in England. Furthermore, the data on adults of all ages, not just older people, identifies the participation characteristics for a general population and provides new insight in those predictors and barriers into social participation. This is strengthened by the data containing information on asset availability for individuals, as this provides a more thorough analysis for this field. This is because we are able to assess the effects of availability whilst controlling for a rich set of individual-, household- and area-level characteristics. We find that having a high concentration of assets locally contributes more to enacting participation over the deprivation level and household income.

The policy implications of this study feeds into existing policies within England and internationally. In terms of social prescribing schemes, this study indicates there should be a focus on encouraging participation in those who live in the most deprived areas and those individuals with lower levels of education. Local infrastructure is important for social participation, and this should continue to be promoted. These assets could include places of worship, sports centres, community centres, youth clubs and libraries. These buildings and assets should be built and protected in areas that have low levels of participation, which tend to be urban and deprived areas in the North of England.

### Limitations of this study

This study used pooled cross-sectional data rather than longitudinal data, meaning the predictors of participation should be interpreted as associations. We were unable to examine the temporal relationship between participation and availability, as we do not know when or for what reason the asset was built. For example, we cannot be sure if there are more places of worship in an area because of demand, or whether the existence of a place of worship makes individuals who live nearby more likely to attend. Additionally, due to using self-reported data on availability of assets there is potential for measurement error. This arises in two ways, firstly for an individual to report an asset is available they firstly must be aware it is there, and secondly, due to the survey asking if it is a 15-20-min walk away, this could be open to ambiguity. We only used responses from the online survey, implying that in order to fill in the survey an individual must have access to a device and internet. This could mean those individuals have the ability to find groups more readily. Finally, the geographical indicators provided by the survey are broad and limit inclusion of further secondary data on reported public spending on community assets and social participation.

## Funding

AW was funded by Wellcome, ref. 217868/Z/19/Z. LAM and MS receive funding from the NIHR Applied Research Collaboration Greater Manchester. MS is an NIHR Senior Investigator. The views expressed are those of the authors and not necessarily the NIHR, the NHS or the Department for Health and Social Care.

## Supplementary Material

Appendix_ddac043

## Notes

**A. Wilding**, PhD Student

**L. A. Munford**, Senior Lecturer in Health Economics

**M. Sutton**, Chair in Health Economics
