# Supplementary material for: Predictors of social participation: evidence from repeated cross-sectional population surveys in England
Source: J Public Health (Oxf). 2022 Mar 17;45(2):379–88. doi: 10.1093/pubmed/fdac029 (PMC10273387; doi:10.1093/pubmed/fdac029)
Supplement: Appendix_ddac043 [file appendix_ddac043.docx]

**Appendix**

Table A.1: Types of groups included in the Community Life Survey

| Name | Description |
| --- | --- |
| Sport/Exercise | Sport/exercise (taking part, coaching or going to watch) (e.g. Sports clubs or groups (e.g. football, swimming, fishing, golf, keep-fit, hiking), Supporter clubs) |
| Local Community | Local community or neighbourhood groups (e.g. Tenants’ / Residents’ Association, Neighbourhood Watch, community group, local pressure group) |
| Religion | Religion (e.g. Attending a place of worship (church, chapel, mosque, temple, synagogue), Attending faith-based groups, Saturday/Sunday School) |
| Hobbies & Arts | Hobbies, Recreation/Arts/Social clubs (e. g. Clubs or groups for the Arts (e.g. theatres, museums, amateur dramatics, orchestras), Hobby or cultural groups (e.g. local history club, Social club) |
| Children's Education | Children's education/schools (e.g. Parent Teacher Associations, School governor, Supporting fairs and fundraising, Helping in school, Running pupils’ clubs) |
| Citizens | Citizens' Groups (e.g. Rotary Club, Lion’s Club, Women’s Institute (WI), Freemasons) |
| Environment | The environment, animals (e.g. National organisations (e.g. Greenpeace, National Trust, RSPCA), Local conservation groups, Preservation societies) |
| Safety & First Aid | Safety, First Aid (e.g. Red Cross, St. Johns Ambulance, Life Saving, RNLI, Mountain Rescue, Helping after a disaster) |
| Education for Adults | Education for adults (e.g. Attending or teaching classes, Mentoring, Cultural groups, Students Union, College governor) |
| Children's Activities | Youth/children's activities (outside school) (e.g. Youth clubs, Sports clubs, Hobby or cultural groups for children) |
| The Elderly | Older people (e.g. Involved with groups, clubs or organisations for older people e.g. Age UK, Pensioner’s clubs, visiting, transporting or representing older people) |
| Justice & Human Rights | Justice and Human Rights (e.g. Special Constable, Magistrate, Legal advice centre, Victim Support, Prison visiting or aftercare, Justice and peace groups, Community or race relations, LGBT groups, National organisations (e.g. Amnesty International)) |
| Politics | Politics (e.g. Membership of, or involvement with, political groups, Serving as local councillor) |
| Health & Social Welfare | Health, Disability and Social welfare (e.g. Medical research charities, Hospital visiting, Disability groups, Social welfare (e.g. Oxfam, NSPCC, Samaritans, Citizens Advice Bureau), Offering respite care, Self-help groups (e.g. Alcoholics Anonymous)) |
| Trade Union | Trade union activity (e.g. Membership of, or involvement with, a trade union.) |

Table A.2: Individual, Household and Area characteristics

| **Characteristics** | **Detail** | **Variable Type** |
| --- | --- | --- |
| **Age** | Categories: | Categorical |
|  | 16 to 19 years |  |
|  | 20 to 24 years |  |
|  | 25 to 34 years |  |
|  | 35 to 49 years (Base Category) |  |
|  | 50 to 64 years |  |
|  | 65 to 69 years |  |
|  | 70 to 74 years |  |
|  | 75 years + |  |
| **Gender** | Female =1 | Binary |
| **Ethnicity** | Black and Asian Minority Ethnicity (BAME) =1 | Binary |
| **Marital Status** | Categories: | Categorical |
|  | Single |  |
|  | Married (Base Category) |  |
|  | Divorced/Separated |  |
|  | Widowed |  |
|  | Not Reported |  |
| **Highest Qualification Obtained** | Categories: | Categorical |
|  | No Qualifications (Base Category) |  |
|  | GCSEs |  |
|  | A Levels |  |
|  | Degree |  |
| **Household Income** | Logarithmic Value | Continuous |
| **House Composition** | Number of Adults | Count |
|  | Number of Children | Count |
| **Housing Tenue** | Homeowner=1 | Binary |
| **Index of Multiple Deprivation** | Categories: | Categorical |
| **Quintile** | 1 - Most |  |
|  | 2 |  |
|  | 3 (Base Category) |  |
|  | 4 |  |
|  | 5 - Least |  |
| **Region** | Categories: | Categorical |
|  | North East |  |
|  | North West |  |
|  | Yorkshire and Humberside |  |
|  | East Midlands |  |
|  | West Midlands |  |
|  | East of England |  |
|  | London |  |
|  | South East |  |
|  | South West |  |
| **Rurality** | Rural=1 | Binary |
| **Survey Year** | Categories: | Categorical |
|  | 2013 (Base Category) |  |
|  | 2014 |  |
|  | 2015 |  |
|  | 2016 |  |
|  | 2017 |  |
|  | 2018 |  |

**Interval regression model for predicting a continuous measure of income**

Income is reported in bands, this means we are not able to equivalise for household composition and adjust for inflation. To get a continuous measure of income, we estimated an interval regression model. This allows us to then to equivalise for the household composition using the Office for National Statistics (ONS) specification, and then deflate it using the consumer price index (CPI) using our first year of the sample (2013) as the base year.

Interval regression models use the upper and lower bands of the categorical outcome to fit where an observation falls in the interval^1^. The structural form of this is as follows:

$$y_{i}^{*}=\boldsymbol{X}_{\boldsymbol{i}}\boldsymbol{\beta}+\epsilon_{i}$$

Where $y_{i}^{*}$ is the latent variable of income, which is interval censored; we only observe which band household income lies in. For our model of income these bands are contained in table A.3. For the upper bound of the top category this is set to missing. The reasoning behind this is that we do not know the maximum income, which makes our model right censored. For our interval model we predict linear income, which the bounds are in table A.3, and logarithmic income where we take the (natural) logarithm of the lower and upper bounds.

Table A.3: Household Income, with lower and upper bands

| **Bands** | **Lower Bound (**$y_{l}$) | **Upper Bound (**$y_{u}$) |
| --- | --- | --- |
| <£5000 | 1 | 4,999 |
| £5000 - £9999 | 5,000 | 9,999 |
| £10000 - £14999 | 10,000 | 14,999 |
| £15000 - £19999 | 15,000 | 19,999 |
| £20000 - £29999 | 20,000 | 29,999 |
| £30000 - £49999 | 30,000 | 49,999 |
| £50000 - £74999 | 50,000 | 74,999 |
| >£75000 | 75,000 | . |

Contained in $\boldsymbol{X}_{\boldsymbol{i}}$ we used a range of socio-economic status variables, area characteristics and interactions between these variables. We assume that the error term,$\epsilon_{i}$, is heteroskedastic and hence use robust standard errors. We use probability weights provided to adjust our sample to the population level.

In order to determine the best fit of the model we use the Akaike information criterion (AIC)^2^ and the Bayesian information criteria (BIC)^3^.

Contained in our $\boldsymbol{X}_{\boldsymbol{i}}$, is the following specification for our preferred model which had the lowest AIC and BIC values. Note - * indicates an interaction between variables.

**Individual characteristics:** Female, Age (Base 35-49) - 16-19, 20-24, 25-34, 50-64, 65-74, Ethnicity - Non-White Ethnicity, Highest Qualification obtained (Base No Qualifications) – GCSEs, A Levels, Degree, Marital Status (Base Married) – Single, Divorced/Separated, Widowed, Missing, Homeowner, Number of Adults*Number of Children in Household

**Area characteristics:** IMD Quintiles (Base IMD=3), Rurality*Government Office Region (Base London) - North East, North West, Yorkshire and Humberside, East Midlands, West Midlands, East of England, South West and South East

**Survey year controls:** (2013 Base), 2014, 2015, 2016, 2017, 2018

Post-estimation of the interval regression model, we predicted income, set between the interval bands, which are contained in table A.3 with $y_{l}=a, y_{u}=b$.

Post predicted outcomes we equivalise for household composition and deflate for inflation.

### *Equivalisation*

The ONS provide the following table for equivalence scale values in table A.4, these are used for equivalising income.

Table A.4: OECD-modified equivalence scale as applied by household composition

| Type of Household Member | Equivalence value |
| --- | --- |
| First adult | 1.0 |
| Additional adult | 0.5 |
| Child aged: 14 and over | 0.5 |
| Child aged: 0-13 | 0.3 |

Source: ONS Equivalised Income^4^

For the linear models, we divide through via the equivalence scale for each predicted income, and for logarithmic model we took the logarithmic value of the equivalence scale and subtracted from the predicted logarithmic income.

*Deflating Income*

We use the consumer price index (CPI) inflation from the ONS to deflate income. We used 2013, the first year of our data, as the base year. Using this we firstly created the “deflator”:

$$deflator_{t}=\frac{CPI_{t=2013}}{CPI_{t}}$$

This value is used to deflate all predicted income, if this was the linear model we multiply through by the deflator, and if this was the logarithmic model, we took the log of the deflator and added to the value. Once completing all these stages, we have our continuous variable for income that we use in our main models, and as a measure of deprivation at the individual level.

### **Missing Values**

In surveys it is not uncommon for individuals to opt to not report their income, this has effects on both on the sample size and non-random missingness. Since we use an interval regression model to predict a continuous variable from a categorical variable, we can use the interval regression estimates to predict the missing values. We do this by first predicting the income of those individuals with non-missing values, $\hat{Y}$.

If we predict the income of the individuals with the upper and lower bands set to missing this means our predicted values can go to infinity and can be negative. These are not possible in real life, and also not intuitive. To prevent this, we set the interval band for those with non-reported income using percentiles of predicted income $\hat{Y}$. This also has benefits of controlling for the distribution of non-missing predictions. We set our interval (a,b), such that:

$$y_{l}=P_{1}\left( \hat{Y} \right), y_{u}=P_{99}\left( \hat{Y} \right)$$

Where $P_{x}$ refers to the xth percentile in the distribution of our predicted income. This method allows use to firstly predict non-negative income, secondly controls for the previous distribution and finally allows us to include those individuals in our models which would have otherwise been omitted. Post estimation we equivalize and deflate for inflation as for the non-missing estimates.

**References**

1. Stewart NF. Interval arithmetic for guaranteed bounds in linear programming. J Optim Theory Appl 1973;12:1–5.
2. Sakamoto Y, Ishiguro M, Kitagawa G. Akaike information criterion statistics. Dordr Neth Reidel 1986;81.
3. Gao X, Song PX-K. Composite Likelihood Bayesian Information Criteria for Model Selection in High-Dimensional Data. J Am Stat Assoc 2010;105:1531–40.
4. Hagenaars AJM, Statistical Office of the European Communities L (Luxembourg) eng, De Vos K, Asghar Zaidi M. Poverty statistics in the late 1980s: research based on micro-data. 1994.URL https://agris.fao.org/agris-search/search.do?recordID=XF2015009737 Accessed 10 June 2020.
